# De novo centromere formation on chromosome fragments with an inactive centromere in maize (*Zea mays*)

**DOI:** 10.1007/s10577-021-09670-5

**Published:** 2021-08-18

**Authors:** Ryan N. Douglas, Hua Yang, Bing Zhang, Chen Chen, Fangpu Han, Jianlin Cheng, James A. Birchler

**Affiliations:** 1 Division of Biological Sciences, University of Missouri, Columbia, MO 65211 USA; 2 State Key Lab of Plant Cell and Chromosome Engineering, Institute of Genetics and Developmental Biology, Chinese Academy of Sciences, Beijing, 100101 China; 3 Department of Electrical Engineering and Computer Science, University of Missouri, Columbia, MO 65211 USA

**Keywords:** CENH3, Centromere reactivation, De novo centromere formation

## Abstract

The B chromosome of maize undergoes nondisjunction at the second pollen mitosis as part of its accumulation mechanism. Previous work identified 9-Bic-1 (9-B inactivated centromere-1), which comprises an epigenetically silenced B chromosome centromere that was translocated to the short arm of chromosome 9(9S). This chromosome is stable in isolation, but when normal B chromosomes are added to the genotype, it will attempt to undergo nondisjunction during the second pollen mitosis and usually fractures the chromosome in 9S. These broken chromosomes allow a test of whether the inactive centromere is reactivated or whether a de novo centromere is formed elsewhere on the chromosome to allow recovery of fragments. Breakpoint determination on the B chromosome and chromosome 9 showed that mini chromosome B1104 has the same breakpoint as 9-Bic-1 in the B centromere region and includes a portion of 9S. CENH3 binding was found on the B centromere region and on 9S, suggesting both centromere reactivation and de novo centromere formation. Another mini chromosome, B496, showed evidence of rearrangement, but it also only showed evidence for a de novo centromere. Other mini chromosome fragments recovered were directly derived from the B chromosome with breakpoints concentrated near the centromeric knob region, which suggests that the B chromosome is broken at a low frequency due to the failure of the sister chromatids to separate at the second pollen mitosis. Our results indicate that both reactivation and de novo centromere formation could occur on fragments derived from the progenitor possessing an inactive centromere.

**Supplementary Information:**

The online version contains supplementary material available at 10.1007/s10577-021-09670-5.

## Introduction

Eukaryotes require a functional centromere for faithful transmission of genetic material during cell division. The centromere is the region of DNA underlying the kinetochore, which is a complex structure of protein and RNA that orchestrates chromosome movement during mitosis and meiosis by serving as a scaffold to attach the chromosome to microtubules (Wang and Dawe 2018). Chromosomes contain only one active centromere, as multiple active centromeres could attach to different spindle poles and rip a chromosome apart during cell division, but exceptions do exist (Zhang et al. 2010) and holocentric chromosomes have attachments along the length of the chromosome (for a review, Bures et al. 2012).

Centromeres typically contain highly repetitive, species-specific DNA sequences (for review: Hartley and O’Neill 2019). Many studies have shown that the DNA sequences found in canonical centromeres are neither required, nor sufficient, to establish kinetochore formation (for review: Burrack and Berman 2012). The DNA sequence of maize (*Zea mays*) centromeres is composed of a 156-bp centromere-specific satellite repeat CentC and Centromeric Retrotransposon of Maize (CRM) family members (Jiang et al. 1996; Ananiev et al. 1998; Wolfgruber et al. 2009). Supernumerary maize B chromosome centromeres have similar DNA components, but CentC and CRM elements are joined by arrays of a B-specific repeat (ZmBs) in the centromeres of the maize B chromosome, which enables easy identification of the B centromere in cytological spreads (Lamb et al. 2005; Blavet et al. 2021).

When specific DNA sequences are unable to form functional centromeres, centromere specification is said to be epigenetic. Several epigenetic marks are associated with active centromeres in plants including the presence of the centromere-specific histone H3 variant CENH3 (CENP-A in mammals) (Zhong et al. 2002), which replaces ~ 15% of canonical H3 nucleosomes within animal and yeast centromeres (Joglekar et al. 2008; Johnston et al. 2010; Bodor et al. 2014), pericentromeric phosphorylation of histone H3 Ser-10 (Manzanero et al. 2000; Houben et al. 2007a, 2007b; Gao et al. 2011), and histone H2A Thr-133 phosphorylation (Dong and Han 2012; Fu et al. 2013).

The maize B chromosome is a supernumerary chromosome that occurs in only some lines and can vary in copy number amongst individuals that carry it. Presence of a low number of B chromosomes is generally neither advantageous nor deleterious, although plants containing many B chromosomes have reduced fitness and/or mild leaf phenotypes such as striping (Staub 1987). Since B chromosomes provide no selective advantage for their host, they require a drive mechanism to maintain themselves in populations. Maize B chromosomes utilize nondisjunction in the second pollen mitosis, and the process requires trans-acting factors located on the long arm of the B chromosome (Roman 1947; Lin 1978; Lamb et al. 2006). The second aspect of the drive mechanism involves preferential fertilization of the egg versus the central cell by the sperm containing the B chromosomes (Roman 1948).

The B-A translocation stock TB-9Sb contains an intact, functional B centromere attached to the short arm of chromosome 9 (9S; Robertson 1984). Through a series of translocations and rearrangements, the active B centromere of TB-9Sb was transferred to the distal end of an otherwise normal 9S (Han et al. 2006). This dicentric chromosome, 9-B inactivated centromere-1 (9-Bic-1), is stable in mitosis and meiosis because the B centromere was inactivated upon transposition (Han et al. 2006). In the presence of a B chromosome, which supplies the trans-acting factors required for nondisjunction, 9-Bic-1 may attempt to undergo nondisjunction (Han et al. 2007). This finding shows that centromere activity is not needed for nondisjunction. The inactive centromere remains adhered together at the second pollen mitosis in this case and thus its short arm occasionally breaks and releases the inactive B centromere when the chromosome 9 centromeres proceed toward opposite poles (Han et al. 2006, 2007).

**Fig. 1:**
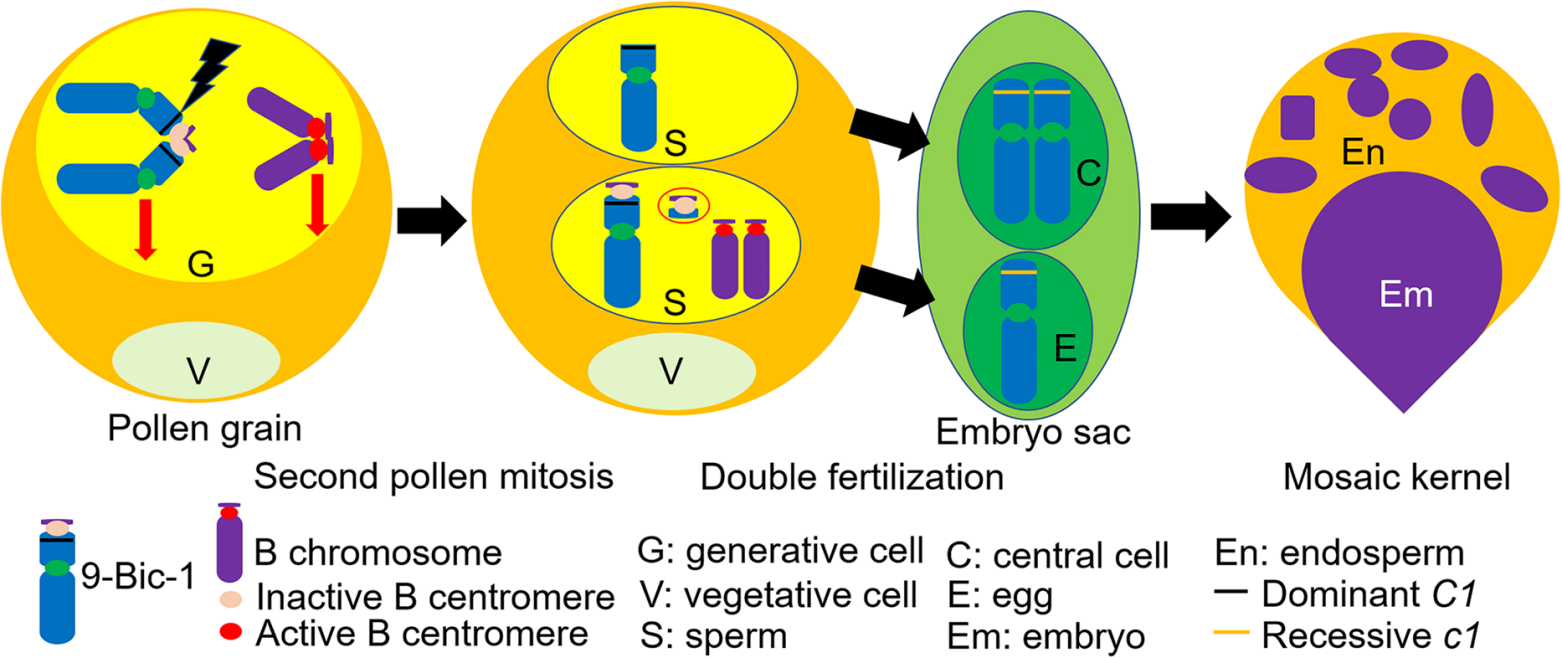
Diagram of 9-Bic-1 breakage during the second pollen mitosis. At the second pollen mitosis, the adhesion of the B centromere on the 9-Bic-1 sister chromatids could break 9S (lightning bolt) when the centromeres of the chromosome 9 sisters proceed toward opposite poles. This will give rise to two sperm cells, with one containing a broken chromosome 9S and with the other containing a chromosome fragment resulting from the breakage. If the breakage happens distal to the *C1* marker gene and the sperm cell with the broken chromosome 9 fuses with the central cell during the double fertilization process, the chromosome 9 with the broken end will initiate a breakage-fusion-bridge (BFB) cycle in the endosperm and show mosaicism for the *C1* gene during endosperm development. The purple section is a group of cells that contains the dominant *C1* allele, while the yellow sectors develop from endosperm cell without *C1*. The mosaic endosperm can be used as the indication of 9-Bic-1 breakage. In most cases, the broken fragment in the embryo will be lost unless it acquires an active centromere via reactivation or de novo formation to make it heritable

We were able to use this property of 9-Bic-1 in conjunction with *C1*, a dominant allele of the *c1* gene required for anthocyanin pigmentation in the embryo and endosperm of the kernel, to identify plants possessing 9-Bic-1 that had undergone chromosome breakage. Plants containing one copy each of 9-Bic-1 and chromosome 9, each containing *C1*, were crossed to a *c1* tester line. If 9-Bic-1 breaks distal to *C1*, the chromatid-type breakage-fusion-bridge (BFB) cycle will occur, and a mosaic color pattern will be seen in the endosperm (Fig. 1). If 9-Bic-1 breaks proximal to *C1*, the resulting BFB cycle will produce a colorless endosperm and colored embryo (Han et al. 2007). The endosperm and embryo will be fully colored if no breakage occurs. The loss or mosaic phenotype of C1 in the endosperm would be associated with embryos that receive the other sperm containing the broken fragment.

Here, we attempted to reactivate the inactive B centromere on 9-Bic-1 by utilizing nondisjunction afforded by the presence of B chromosomes. We identified two heritable mini chromosomes containing released B centromeres. One of the mini chromosome arose from 9-Bic-1 chromosomal breakage, and the second mini chromosome originated via B chromosome breakage. Chromatin immunoprecipitation-sequencing (ChIP-seq) showed that the released centromere in 9-Bic-1 derived mini chromosome regained association with detectable CENH3 together with the establishment of a de novo centromere. For the second mini chromosome, the B centromere originating from the B chromosome lost function, but a de novo centromere arose elsewhere on the chromosome.

## Materials and methods

### Plant material

All lines used in this study were in the B73 inbred background. Seedlings were grown in the Ernie and Lottie Sears Plant Growth Facility at the University of Missouri-Columbia under the following conditions: 16 h light, 25 C day/20 C night.

### FISH and immunolocalization

Fluorescence in situ hybridization (FISH) and immunolocalization in mitosis and meiosis were performed as described (Kato et al. 2004; Han et al. 2009; Gaeta et al. 2011). A combination of 5′-labeled synthetic oligonucleotides and nick-translated probes were used. CentC was labeled with 6-FAM (fluorescein; green); telomeric sequences, which hybridize to ZmBs (Alfenito and Birchler 1993; Masonbrink et al. 2012), were labeled with 6-FAM or Texas Red. The signal from the B centromere at the exposure used is readily visible, while the telomeres are not. ZmBs was labeled with Texas Red. Individual loci from 9S were amplified from B73 genomic DNA (see Supplemental Table 2) and labeled with Texas Red-dCTP by nick translation (Kato et al., 2004). Chromosomes were counterstained with 4′,6-diamidino-2-phenylindole (DAPI) and mounted with Vectashield (Vector Laboratories). Images were captured on an Olympus BX61 microscope using Applied Spectral Imaging Software V.4.5-SP3 and a COOL-1300QS digital camera.

### Copy number variation analysis

CNV analyses were performed as described previously (Yang et al. 2021). In brief, genomic DNA sequencing data were trimmed using cutadapt (Martin 2011) and filtered with FASTX toolkit (Hannon 2010) using parameter -Q33 -q 20 -p 80. Then, each fastq file was aligned to B73 reference v4 (Jiao et al. 2017) plus mitochondria and chloroplast genomes using Bowtie2 (Langmead and Salzberg 2012) under parameter –phred33 -N 0 –no-unal -k 10. The SAM file was further filtered to keep 0 mismatch reads using a perl script. Only unique mapped reads in the SAM file were kept using a perl script. The read number within each gene region was counted for each mini B, 9-Bic-1 or B73. Ratios were calculated by comparing mini B or 9-Bic-1 to B73 and plotted along each chromosome using R.

### B chromosome deficiency mapping

Deficiency mapping to mini chromosomes was performed as described previously (Blavet et al. 2021). In brief, low-quality read filtering and mapping procedures are the same as in the copy number variation analysis. The uniquely mapped reads that have 0 mismatches in the SAM file were regarded as aligned reads on the A chromosomes and were filtered out from the fastq (after filtering low quality reads) file using a *perl* script to obtain the unmapped reads. Each fastq file that consists of unmapped reads was aligned to the B chromosome (Blavet et al. 2021) and only reads that mapped to a unique location were kept. The reads were counted in the regions of 1 kb along each sequence and the results were plotted by ggplot2 in R.

### ChIP-seq and data analysis

CENH3 ChIP-seq was performed as described (Fu et al. 2013) using ~ 5.0–7.5 g of 14-day whole seedling tissue. ChIP DNA was sequenced with paired-end 100 nt Illumina. Reads were trimmed and cleaned with TRIMMOMATIC (Bolger et al., 2014) (LEADING:30 TRAILING:29 SLIDINGWINDOW:4:30 MINLEN:29 AVGQUAL:28) prior to mapping.

For testing CENH3 peaks on A chromosomes (from chromosomes 1 to 10), CENH3 ChIP-seq reads were mapped to the B73 version 4 plus B chromosome using bowtie2 with default parameters. Reads were further filtered to 0 mismatches and unique mapped reads using perl scripts. The alignments were converted to BAM files, then sorted and merged using SAMtools (Li et al. 2009). Read coverage and enrichment were displayed after converting BAM files to TDF files using igvtools (Thorvaldsdóttir et al. 2013).

For CENH3 binding on the B centromere region, we first adopted the approach of B chromosome deficiency mapping to identify B chromosome specific CENH3 ChIP-seq reads. The reads were aligned to the B73 genome with nondefault parameters: “–phred33 -N 0 –no-unal -k 10”; the reads recorded in the SAM file were treated as mapped reads on the A genome and thus filtered out of the original FASTQ file using a perl script and the remaining reads were aligned to the maize B genome (chrB + 307 B scaffolds) using Bowtie2 with nondefault parameters: “–phred33 -N 0 –no-unal.” Only reads that mapped to a unique location on the B genome were kept. The alignments were converted to BAM files, then sorted and merged using SAMtools. Read coverage and enrichment were displayed after converting BAM files to TDF files using igvtools.

## Results

### Identification of mini chromosomes potentially derived from 9-Bic-1

**Fig. 2:**
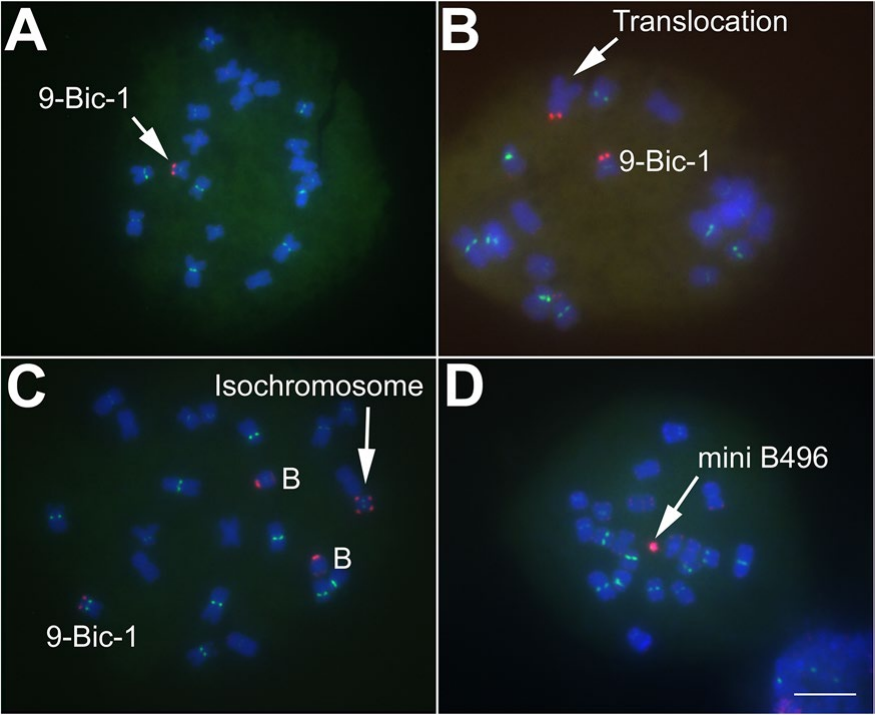
Fates of broken chromosomes. Mosaic kernels that show an indication of breakage of 9-Bic-1 were examined by FISH. Telomere sequences (Telo) are shown in red and CentC, which hybridizes to centromeres, is shown in green. **A** At the second pollen mitosis, breakage occurred on one 9-Bic-1 due to attachment of two sister chromatids, but the broken fragment is lost due to absence of an active centromere. Only the unbroken 9-Bic-1 chromosome is present. **B** The broken chromosome piece was translocated onto another unknown chromosome indicated by a white arrow. **C** 9-Bic-1 underwent mis-division of the centromere of chromosome 9 and fused at the site of centromere breakage to form an isochromosome (white arrow). B chromosomes are denoted as B. **D**, A mini chromosome with intense B repeat was found and could result from breakage of 9-Bic-1 or the B chromosome. Blue depicts chromosomes counterstained with DAPI. Bar equals 10 µm

Approximately 10,000 kernels were screened for breakage of 9-Bic-1, of which 1412 kernels exhibited signs of 9-Bic-1 breakage using the *C1*-based system described above. When 9-Bic-1 is broken, there are four possible fates for the resulting fragment: (i) the B centromere remains inactive, in which case the fragment will be lost (Fig. 2A); (ii) the fragment translocates onto another chromosome that contains an active centromere (Fig. 2B); (iii) centromere mis-division of chromosome 9 centromere of 9-Bic-1 forms an isochromosome that contains an inactive B centromere on both ends (Fig. 2C); or (iv) a broken fragment from either breakage of 9-Bic-1 or the B chromosome becomes heritable (Fig. 2D). FISH was used to examine each of the 1412 kernels that showed signs of chromosome breakage. In the vast majority of cases (*n* = 1391), no fragment was observed, presumably due to loss of the fragment. However, isochromosomes were observed ten times, a single translocation of the inactive B centromere was observed, and ten mini chromosomes containing a B centromere were recovered (Supplemental Table 1; Supplemental Fig. 1; Blavet et al. 2021).

### The B sequences on mini B1104 and mini B496 are different

**Fig. 3:**
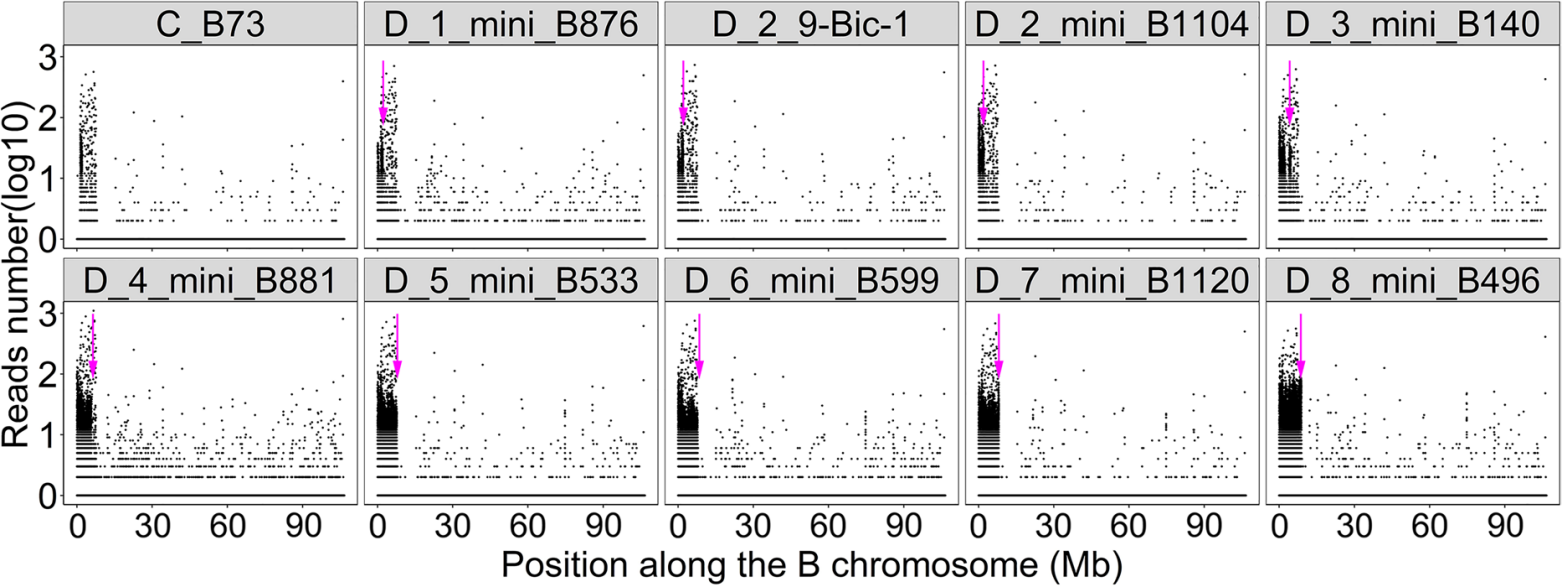
Breakpoints of mini B1104 and mini B496 on B chromosome. Eight mini B chromosomes and 9-Bic-1 were subjected to B chromosome deficiency mapping as previously performed (Blavet et al 2021). B73 without B chromosomes was used as a negative control (C_B73). Sequence reads unique to the B chromosome were aligned to the B chromosome reference sequence. The Y axis is the aligned read numbers of log10 of 1 kb regions on the B chromosome. Each black dot equals 1 kb resolution. The X axis is the position along the B reference genome. Mini B1104 has the same breakpoint as 9-Bic-1 at 2.1 Mb, which is marked by the magenta arrow. The mini B496 is broken at 7.5 Mb, which suggests it is not a 9-Bic-1 derivative. The background signals in B73 and the minichromosomes result from cross homology of knob and CentC from the A chromosomes to the B reference (Blavet et al., 2021). C, control. D, deficiency

Previous research determined the breakpoint of six out of ten mini chromosomes from this screening on the B chromosome by using B chromosome deficiency mapping (Blavet et al. 2021). In that study, mini B876 was found to be broken in the B centromere region, which indicates it could be derived from B centromere misdivision that occurs at a low frequency (Blavet et al. 2021; Carlson and Chou 1981; Kaszás and Birchler 1996; 1998). However, its breakpoint in the centromere could alternatively indicate that it results from 9-Bic-1 breakage followed by reactivation. Five other mini chromosomes showed breakpoints in the proximal knob region likely due to B chromosome breakage (Blavet et al. 2021), given that these sequences are not present in 9-Bic-1. Mini B524 and mini B1139 were not subjected to this analysis because mini B524 material could not be further perpetuated and B chromosomes still exist in mini B1139 material, which makes it unsuitable for deficiency mapping. The results showed that mini B1104 has the same breakpoints as 9-Bic-1 in the centromere and chromosome arm 9S with its B portion broken at the centromere region adjacent to the centromeric knob, which suggest that mini B1104 was a 9-Bic-1 derivative (Fig. 3, Supplemental Fig. 2). The mini B496 might have been derived from an intact B chromosome as it is broken at the B centromeric knob region, which is distinct from 9-Bic-1 (Fig. 3, Supplemental Fig. 2).

### Both mini B1104 and mini B496 contain a 9S segment

**Fig. 4:**
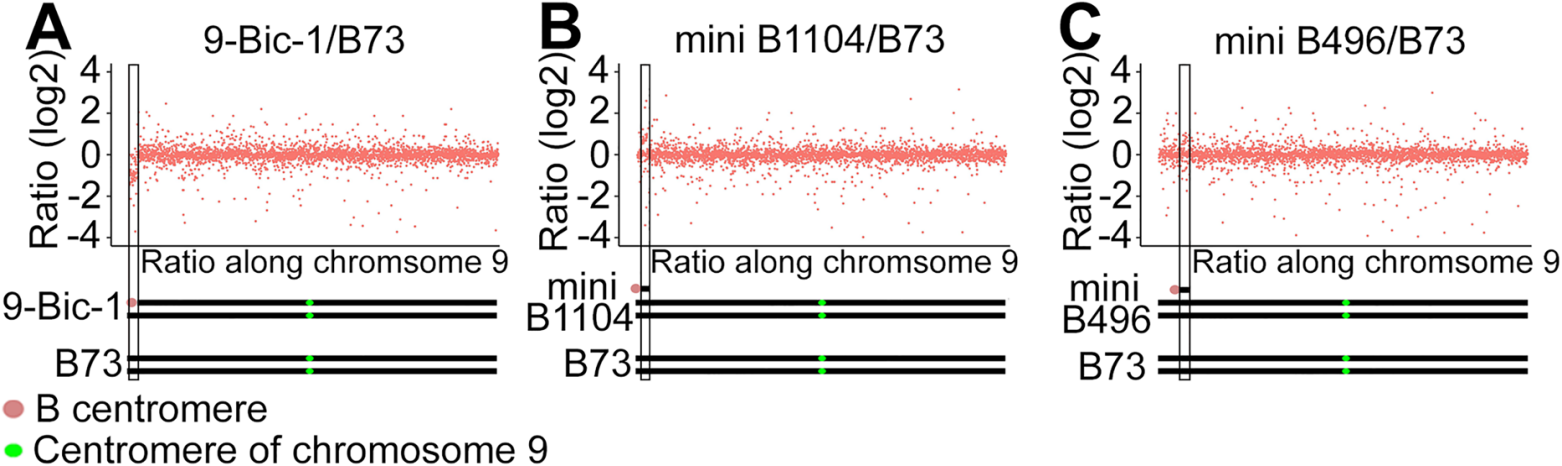
Translocation point of 9-Bic-1 and mini chromosomes on chromosome 9. Copy number variation along chromosome 9 in 9-Bic-1, mini B1104, and mini B496. Y axis is log2 of the ratio. X axis is the ratio along chromosome 9. Each red dot is the ratio comparing read numbers of 9-Bic-1, mini B1104, or mini B496 to B73 within a single gene window. The diagram below each plot shows the chromosome constitution for each group. Red oval represents the inactive B centromere. The centromere of chromosome 9 is depicted as a green circle. The 9-Bic-1 heterozygote contains one 9-Bic-1 and one intact chromosome 9. Both mini B1104 and mini B496 contain one mini chromosome and two chromosomes 9. **A** The first three Mb (box in **A**) showed lower copy number suggesting the breakpoint of 9-Bic-1 is at ~ 3 Mb. **B**, A ~ 500 kb region (box in **B**) immediately after the breakpoint of 9-Bic-1 was found to have a higher ratio in mini B1104 suggesting it is a 9-Bic-1 derivative. **C** A ~ 200 kb region within the ninth Mb in chromosome 9 was found to have a higher ratio in mini B496 (box)

Previous results suggest that 9-Bic-1 lost its chromosome 9 short arm tip because homozygous 9-Bic-1 exhibits an albino phenotype (Han et al. 2007). Plants homozygous for 9-Bic-1 were used to roughly map the translocation point of the inactive B centromere between 1.85 and 3.44 Mb on 9S (Supplemental Fig. 3). To determine the translocation point of 9-Bic-1 accurately, we used genomic DNA sequencing data of a 9-Bic-1 heterozygote and B73 to perform copy number variation (CNV) analysis for chromosome 9. We found the first three Mb on chromosome 9 was approximately at a ratio of 0.5 but the ratio reached 1 from the fourth Mb through the remainder of chromosome 9, which suggests that the breakpoint is located between the 3rd and 4th Mb on chromosome 9. Finally, the breakpoint of 9-Bic-1 was further localized between Zm00001d044806 and Zm00001d044808 (2.94 ~ 3.06 Mb) (Fig. 4).

Next, we checked the CNV of mini B1104 and mini B496 on chromosome 9. The chromosome portion between Zm00001d044808 and Zm00001d044815 (3.06 ~ 3.52 Mb) had a higher ratio compared to the remainder of the region when comparing mini B1104 and B73, which suggests that this ~ 500 kb 9S segment is on mini B1104 (Fig. 4). This result also supports the conclusion that the mini B1104 was directly derived from 9-Bic-1.

Although the centromere of mini B496 likely has an origin from the normal B chromosome, it also contains an interstitial 9S region between Zm00001d044995 and Zm00001d044999 (9.51 ~ 9.67 Mb) because this portion of the chromosome has higher CNV when comparing mini B496 to B73 (Fig. 4). FISH with a probe specific to this region confirmed the presence of a 9S segment in mini B496 (Supplemental Fig. 4). These results suggest that mini B496 might have fused with a broken 9-Bic-1, although its precise history can only be hypothesized. The CNV analysis of the other mini chromosomes indicates that they contain no 9S portion (Supplemental Fig. 5).

### mini B1104 and mini B496 have functional centromere activity

**Fig. 5:**
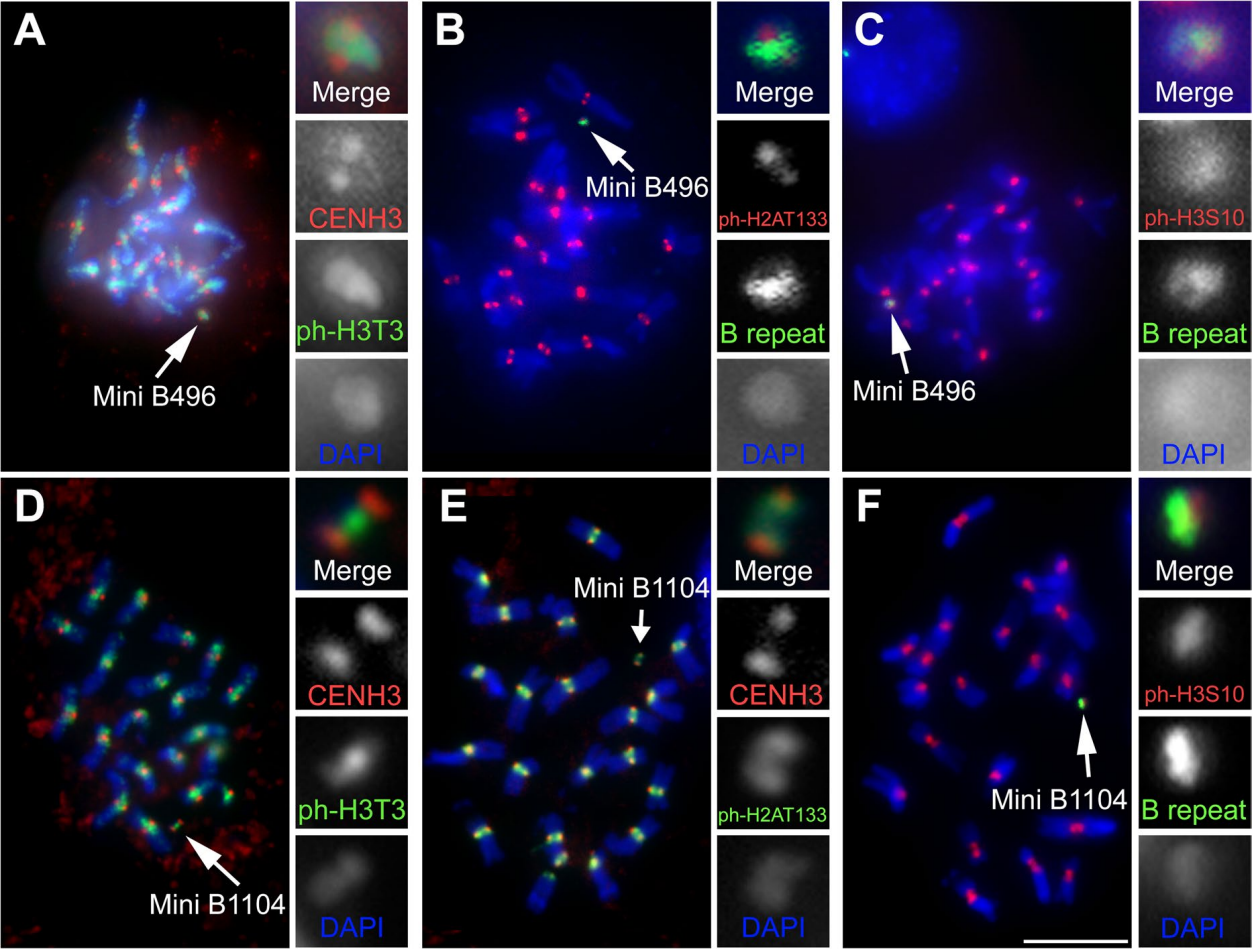
Immuno-FISH of mini B496 and mini B1104. Immunostaining of mini B496 (**A**, **B**, **C**) and mini B1104 (**D**, **E**, **F**) using centromere-specific antibodies including CENH3, phosphorylated H3Thr3, phosphorylated H3Ser10, and phosphorylated H2AThr133. ZmBs (B repeat) was probed with green in **B**, **C**, **F**. The antibody signals on both mini chromosomes confirm the presence of a functional centromere. Arrows indicate the mini chromosome. Insets display an enlarged image of mini B496 and mini B1104 in each panel. Blue depicts chromosomes counterstained with DAPI. Scale bar, 10 µm

The two mini chromosomes, mini B496 and mini B1104, have been inherited through at least seven generations, which indicates that they contain a functional centromere. Immunolocalization showed that known epigenetic marks of functional maize centromeres, such as CENH3 (Zhong et al. 2002), H3-Ser10ph (Gao et al. 2011), and H2A-Thr133ph (Dong and Han 2012), are present on both mini B496 and mini B1104 (Fig. 5). Both mini chromosomes also exhibit typical mini chromosome behavior during meiosis (Supplemental Fig. 6; Birchler and Han 2013); they do not pair during pachytene when present in multiple copies (Supplemental Fig. 6A and B), and sister chromatids prematurely separate and lag in anaphase I (Supplemental Fig. 6E and F).

### *Examination of mini chromosomes reveals *de novo* centromere formation*

In order to determine the DNA sequences associated with the functional centromeres of mini B496 and mini1104, ChIP-seq was performed with a maize anti-CENH3 antibody on three biological replicates each of the inbred B73, B73 with B chromosomes (B73 + B), and lines containing TB-9Sb, 9-Bic-1, and mini B496. Five biological replicates, two from generation 6 and three from generation 7, were subjected to ChIP for line mini B1104. The B73 + B and TB-9Sb lines have a functional B centromere, which can be used as positive controls to test CENH3 binding on the B centromere, while 9-Bic-1, which contains an inactive B centromere, and B73 without B chromosomes can serve as negative controls.

**Fig. 6:**
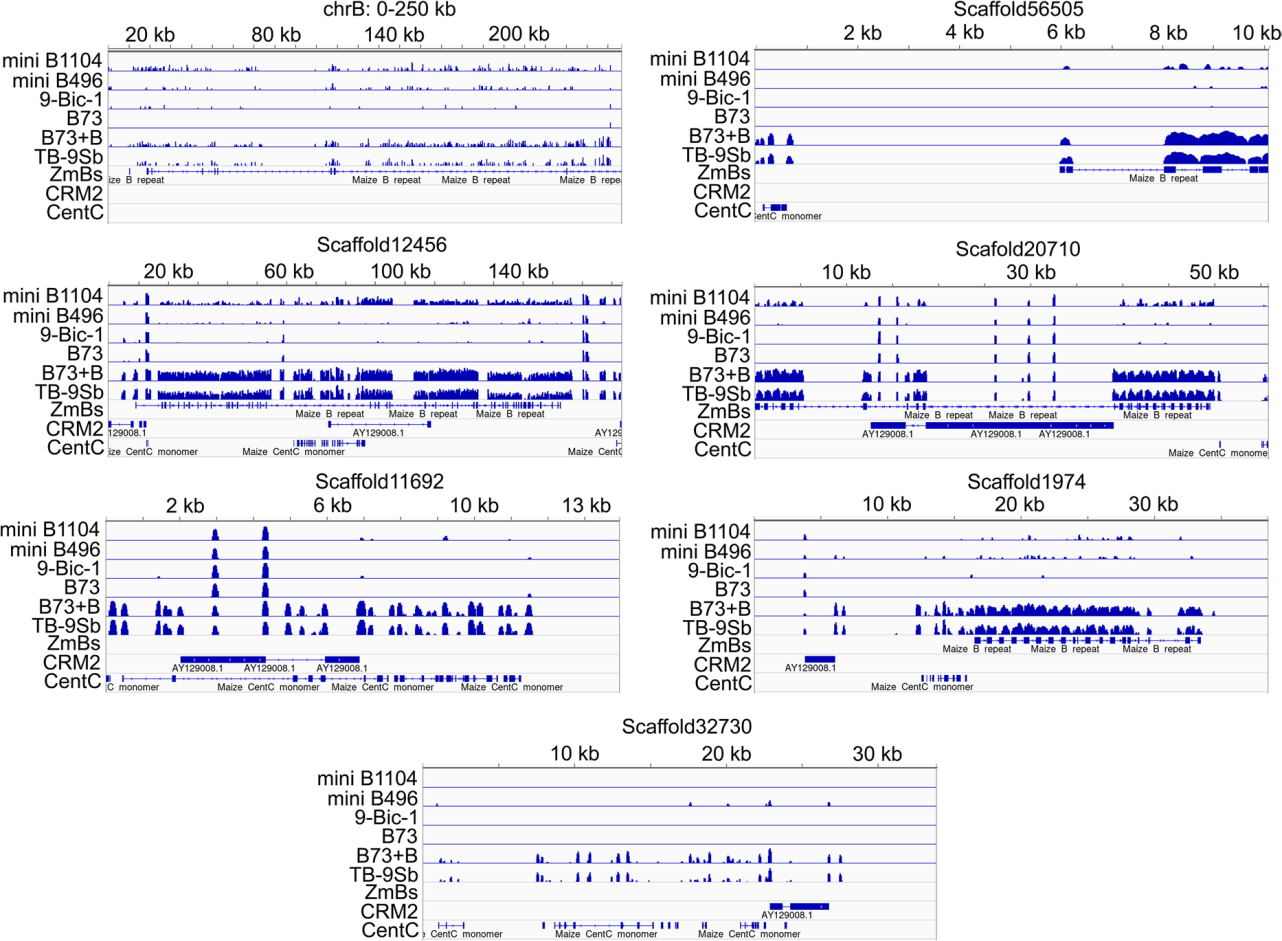
CENH3 binding on B centromere. CENH3 ChIP-seq reads from six samples were mapped to seven B centromeric sequences to determine CENH3 binding. The location of the three most common repeats including ZmBs, CRM2, and CentC are displayed in each scaffold. B73 + B and TB-9Sb each contain an active B centromere, while no B centromere is present in B73. 9-Bic-1 comprises inactive B centromeric chromatin. In B73 plus B and TB9-Sb, CENH3 enrichment was found in each scaffold. For the two mini chromosomes, mini B1104 and mini B496, the CENH3 occupation was found to be remarkably reduced in mini B1104 and almost absent in mini B496. Three kinds of B centromeric repeat, CentC, CRM2, and ZmBs were blasted to each sequence to locate each repeat type in the B centromeric scaffolds

The B chromosome sequencing project has identified 575 kb of B chromosome centromeric sequences consisting of six B scaffolds and the 250 kb proximal portion of the short arm of the assembled B reference, which includes a portion of the centromere (Blavet et al. 2021). Because the two mini chromosomes contain intact B centromeric regions, we first checked the CENH3 occupation on these sequences. To this end, the CENH3 CHIP-seq reads were first mapped to B73. The reads that aligned to the B73 genome were filtered and the remaining reads were mapped to the maize B segment. This step is performed to reduce mapping ambiguity due to homology of centromeric repeats between the B chromosome and A chromosomes, such as CentC and CRM (Lamb et al. 2005; Jin et al. 2005). When the CENH3 read pileup was compared across all lines in the 575 kb B centromeric sequences (Blavet et al. 2021), it was found that 9-Bic-1 and mini B496 had no or markedly reduced CENH3 binding compared to TB-9Sb and B73 + B that contain active B centromeres (Fig. 6). In contrast, the B1104 centromere sequences showed an association with CENH3 to some degree. These results suggest that reactivation of canonical centromeric chromatin occurred in mini B1104, while for mini B496, whose centromere is derived from the B chromosome, the B centromere became inactive.

**Fig. 7:**
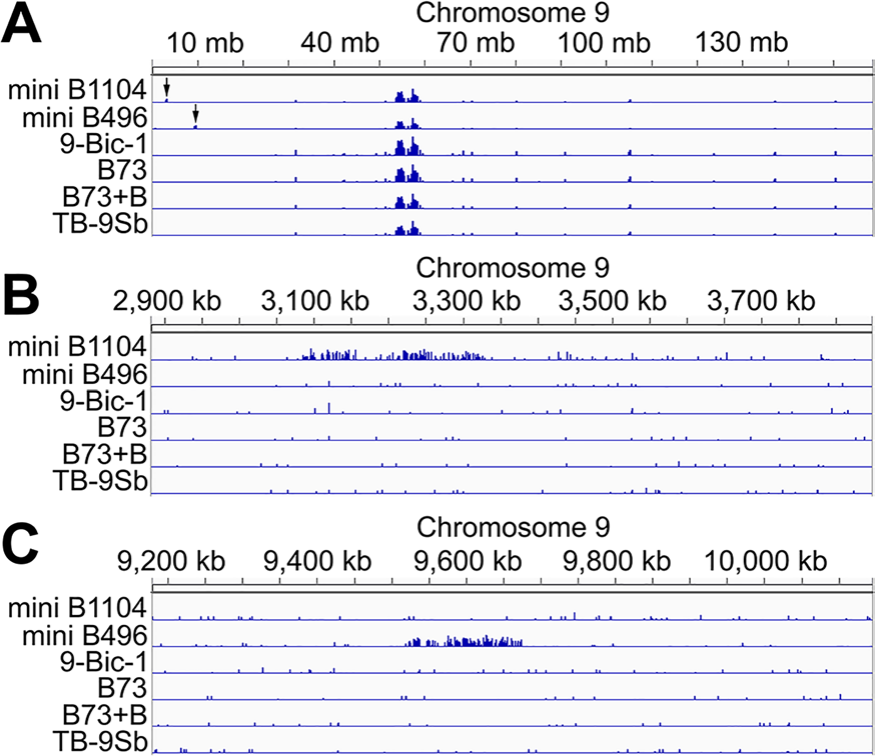
CENH3 binding on chromosome 9. CENH3 reads were aligned to the B73 genome and CENH3 pileups were investigated on 9S. **A** CENH3 binding on chromosome 9. In **B**, a 500 kb CENH3 binding region, which is present on mini B1104, was found on mini B1104 on 9S (3.06–3.52 Mb). **C** in mini B496, the 9S portion (9.51–9.67 Mb), which is included on this mini chromosome, was occupied by CENH3. These results suggest that de novo centromere formation occurred on mini B1104 and mini B496

To determine whether a region other than the canonical centromeric repeats acquired centromere activity, CENH3 enrichment on the A chromosomes was analyzed. The CENH3 binding regions, denoting the centromeres of chromosomes 1 through 10, were virtually indistinguishable amongst the four lines studied (Supplemental Fig. 7). However, lines mini B496 and mini B1104 displayed CENH3 binding peaks on 9S that were absent in all of the other lines (Fig. 7A). In mini B1104, a ~ 300 kb 9S region (from 3.1 to 3.35 Mb) displayed unique CENH3 binding (Fig. 7B). In contrast, for mini B496, the unique CENH3 binding domain stretched for ~ 160 kb from 9.51 to 9.67 Mb on 9S, which is present on this mini chromosome (Figs. 4 and 7C, Supplemental Fig. 4). Taken together, the CENH3 ChIP-seq results strongly suggest that both mini chromosomes contained de novo formed centromeres.

## Discussion

### *Both mini B1104 and mini B496 have *de novo* centromere formation*

Cases of centromere formation in the last two decades show the commonality of de novo centromere formation. Adding alien chromosomes to wheat or oat resulted in chromosomal mutations such that these alien chromosomes lacked the canonical centromeric sequences but still showed normal transmission, which suggested they had de novo centromeres (Nasuda et al. 2005; Topp et al. 2009). Also, many cases of de novo centromeres have been documented in maize (Fu et al. 2013; Liu et al. 2015, 2020; Schneider et al 2016). Our data suggest that a 300 kb region of 9S on mini B1104 gained CENH3 binding. During the formation of mini B496, the active B centromere lost its function but newly active centromeric chromatin formed on non-canonical centromeric sequences of this mini chromosome.

### Centromere reactivation occurred on mini B1104

Previous results documented a small, inactivated B chromosome centromere reactivated after being recovered from an unstable dicentric (Han et al. 2009). However, the result could not discriminate whether exactly the same sequences were associated with function in the original active and reactivated centromeres. Our result suggesting CENH3 binding on the B centromere sequences in mini B1104 indicates the same sequences that were previously inactive can regain CENH3 association. Given the small size of mini B1104, it is not possible to determine whether the B centromere, the de novo CENH3 associated sequences, or both, can organize a kinetochore.

### Mini B496 is a secondary derivative of 9-Bic-1

**Fig. 8:**
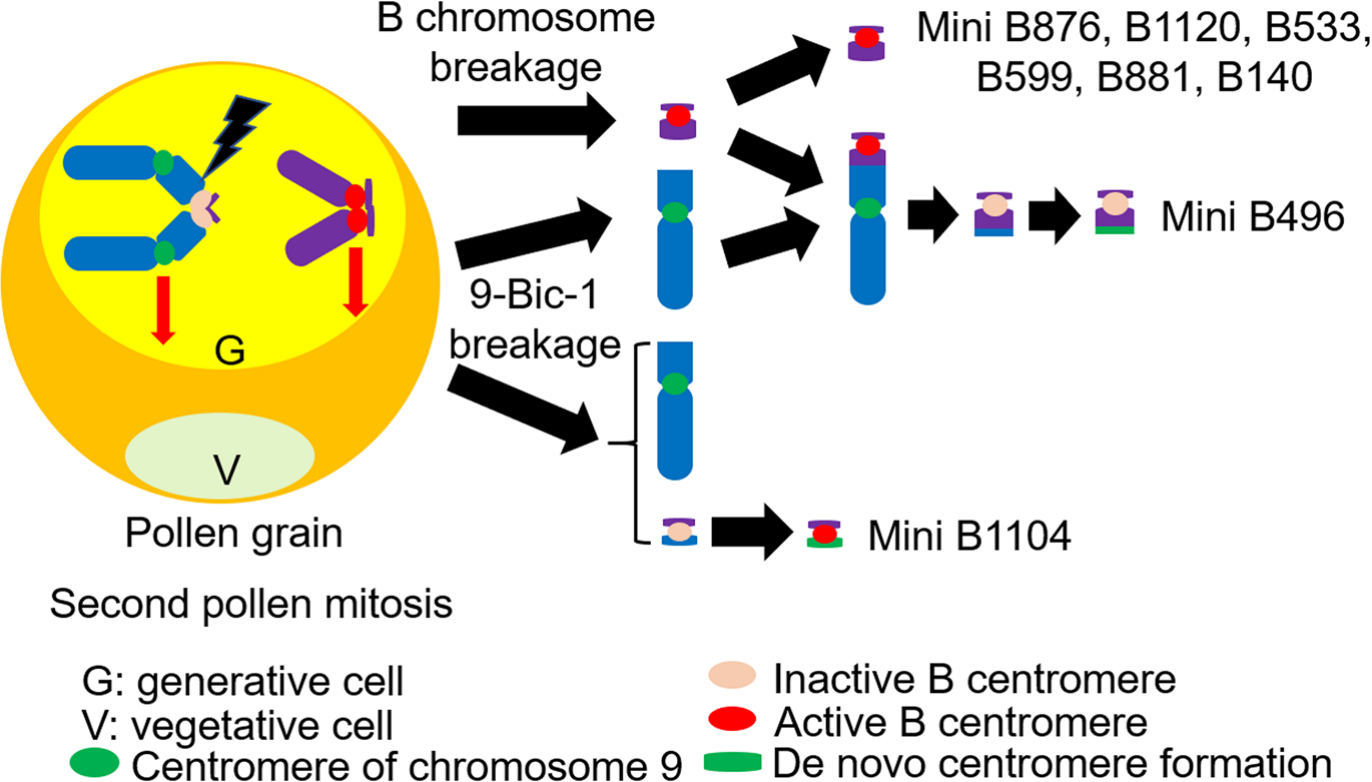
Diagram of origin of mini chromosomes. Breakpoint identification of eight mini chromosomes showed that the centromere chromatids could undergo breakage of 9-Bic-1 and the B chromosome at the second pollen mitosis. Among these mini chromosomes, six of them were derived from the B chromosome. Only mini B1104 was derived from 9-Bic-1. mini B496 is derived from the B chromosome, but also fused with portions of 9S. Centromere activity assays showed that de novo centromere formation and reactivation of the B centromere occurred on mini B1104. In mini B496, the B centromere is inactive but was salvaged by a de novo centromere formation

Based on our analysis, mini B496 is probably derived from a normal B chromosome breakage but it also contains an interstitial fragment from chromosome 9S. It has lost centromere activity on the canonical B centromeric repeats but acquired a de novo formation of a centromere, which allowed it to be inherited. The formation of mini B496 cannot be known with certainty, but might have occurred as follows: (i) a mini B was produced from breakage that happened on a normal B chromosomes during attempted non-disjunction at the second pollen mitosis and its B centromere remained active; (ii) within the same sperm was a fractured chromosome in 9S from an attempted nondisjunction of 9-Bic-1; (iii) the mini B and broken 9-Bic-1 chromosome fused together to form a dicentric chromosome with centromere 9; (iv) the dicentric chromosome fractured on 9S and the B centromeric region of mini B496 lost its function, but de novo centromere formation occurred on the 9S portion (Fig. 8). Previous results have documented the rapid establishment of de novo centromeres in maize (Liu et al. 2020).

### B chromosome breakage during the second pollen mitosis

During the second pollen mitosis, chromosome breakage can occur during non-disjunction of the B chromosome, and such breakage could result in the formation of mini B chromosomes. The broken end of a mini B chromosome will be repaired and stabilized by adding a telomere to its end in the embryo after the sperm with mini B chromosome joins with the egg cell (McClintock 1939; McKnight and Shippen 2004). The inactive B centromere on 9-Bic-1 remains adhered together when normal B chromosomes are present at the second pollen mitosis (Han et al. 2007). Chromosome breakage of 9-Bic-1 is indicated when a mosaic endosperm occurs, but this process does not monitor chromosome breakage on the normal B chromosome.

In this study, 10 mini chromosomes with B repeat signal (as observed using FISH) were recovered from a total of 1412 kernels, which exhibited signs of chromosome breakage in the endosperm. From these 10 mini chromosomes, two of them were not subjected to further breakpoint analysis on the B chromosome due to either a failure to perpetuate the line or because a B chromosome was still present with the mini chromosome, which obscures the ability to perform a comparison with the B reference sequence. From the analysis of the remaining eight mini chromosomes, it is ambiguous as to whether mini B876 was derived from a B centromere mis-division or a similar break in 9-Bic-1 followed by reactivation because its breakpoint was found in the B centromere region.

However, mini B1104 has been definitively determined to be derived from 9-Bic-1. The other six mini chromosomes have breakpoints in the centromeric knob region, which suggests that these mini chromosomes are likely from normal B chromosome breakage. In this case, the percentage of B chromosome breakage is at least 0.42% (6/1412) but potentially an underestimate due to the inability to recognize all cases. This result suggests that the normal B chromosome centromere can be attached to the spindle from both poles at the second pollen mitosis at least in some cases, which can fracture the centromere or the adjacent knob, which is surrounded by the B specific repeat that is involved with nondisjunction (Lamb et al. 2005; Blavet et al. 2021) if the B specific repeat region remains adhered between sister chromatids.

## Supplementary Information

Below is the link to the electronic supplementary material.

Supplementary file1 (DOCX 33708 KB)

## Data Availability

Raw data of B73, 9-Bic-1 and six lines with mini B chromosome used for B chromosome deficiency mapping or CNV analysis were downloaded from NCBI-SRA as BioProject PRJNA634743. Genomic sequencing data of mini B496 and B1104 CENH3 and ChIP-seq data are available in NCBI-SRA as BioProject PRJNA736453.

## Abbreviations

9-Bic-1: 9-B inactivated centromere-1
9S: Short arm of the chromosome 9
BFB: Breakage fusion bridge
CENH3: Centromeric histone H3
ChiP-seq: Chromatin immunoprecipitation-sequencing
CNV: Copy number variation
CRM: Centromeric retrotransposon of maize
DAPI: 4’,6-diamidino-2-phenylindole
FISH: Fluorescence in situ hybridization
