# Supplementary material for: De novo centromere formation on chromosome fragments with an inactive centromere in maize (Zea mays)
Source: Chromosome Res. 2021 Aug 18;29(3-4):313–25. doi: 10.1007/s10577-021-09670-5 (PMC8710440; doi:10.1007/s10577-021-09670-5)
Supplement: Supplementary file 1 — Supplementary file1 (DOCX 33708 KB) [file 10577_2021_9670_MOESM1_ESM.docx]

**
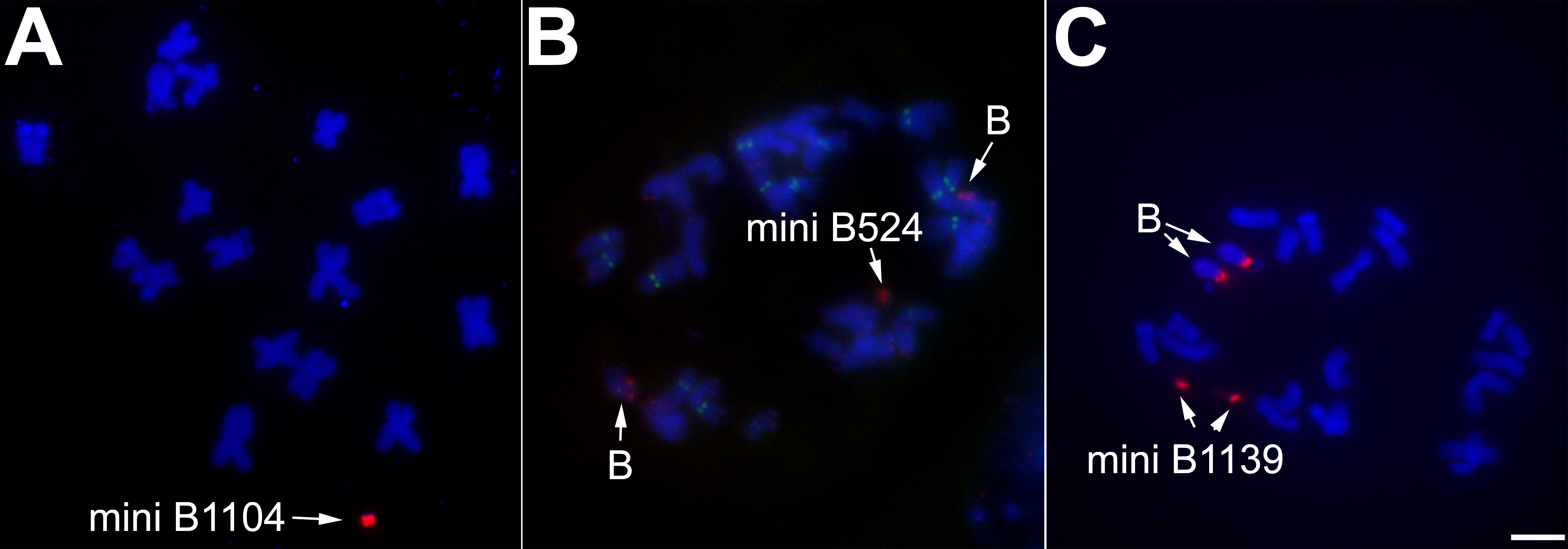
Supplemental Figure 1.** FISH identification of mini 524 and mini 1139.

Plants contain one mini B524 and two B chromosomes (**B**) were found using telomere (Telo) repeat probe (red) (which cross hybridizes with the B centromere) and CentC (green) in **B**. In **A** and **C**, only ZmBs (B repeat) was probed to visualize mini B1104 (**A**) and B1139 (**C**). White arrows indicate mini chromosomes or normal B chromosomes. Bar equals 5 µm.


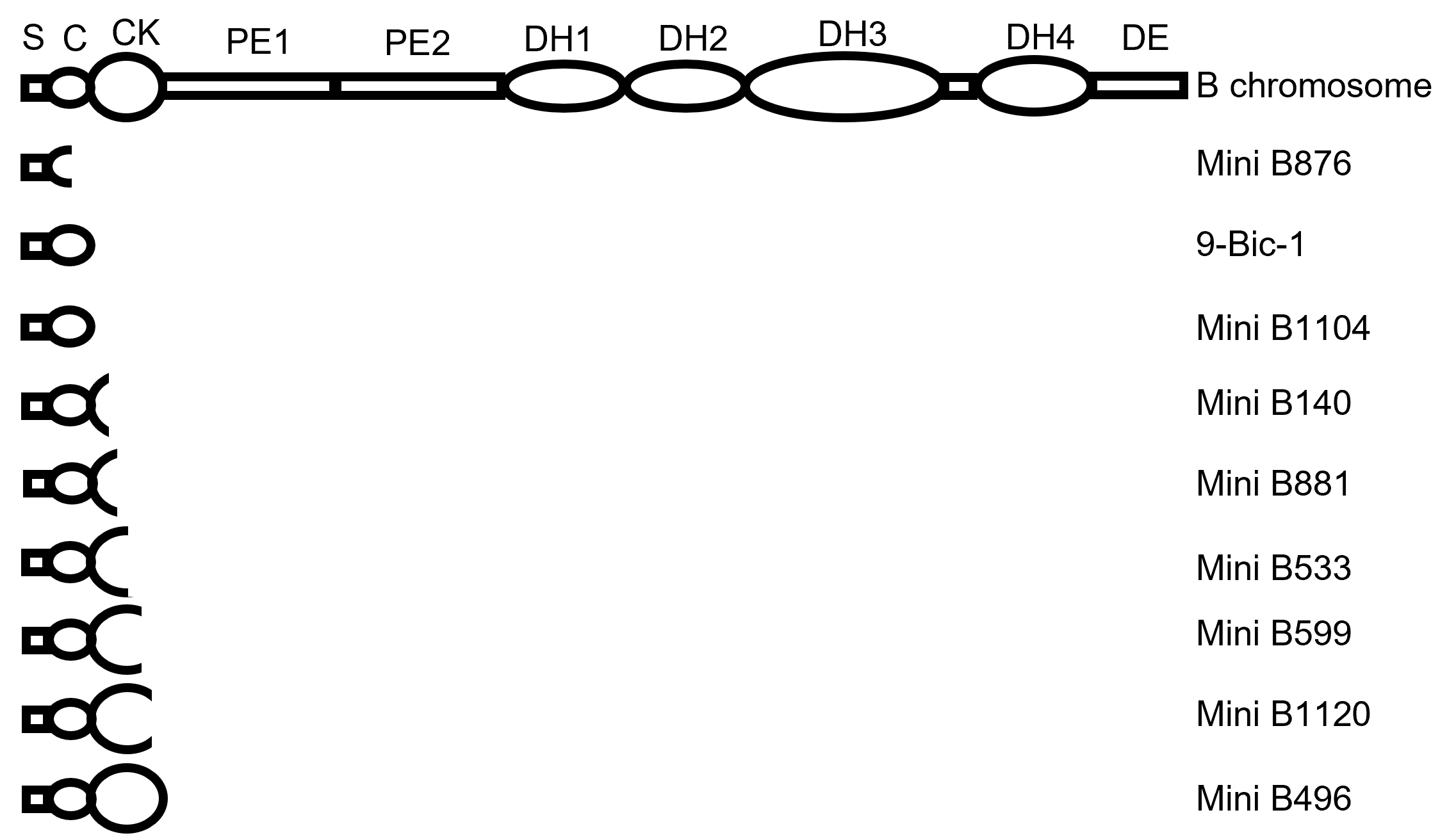
**Supplemental Figure 2.** Diagram of mini B chromosomes.

The B chromosome consists of a short arm (S), a centromere (C), and a long arm composed of a centromeric knob (CK), an euchromatic region (PE), a long heterochromatic region consisting of four blocks (DH), and finally a short distal euchromatic region (DE). A previous publication (Blavet et al. 2021) has documented the information of the breakpoint information of 9-Bic-1 and mini B chromosomes except mini B1104 and B496. Mini B876 breaks at the centromeric region. 9-Bic-1 and mini B1104 have same breakpoint between the centromere and the centromeric knob region. The remainder of the chromosomes break in the centromeric knob.


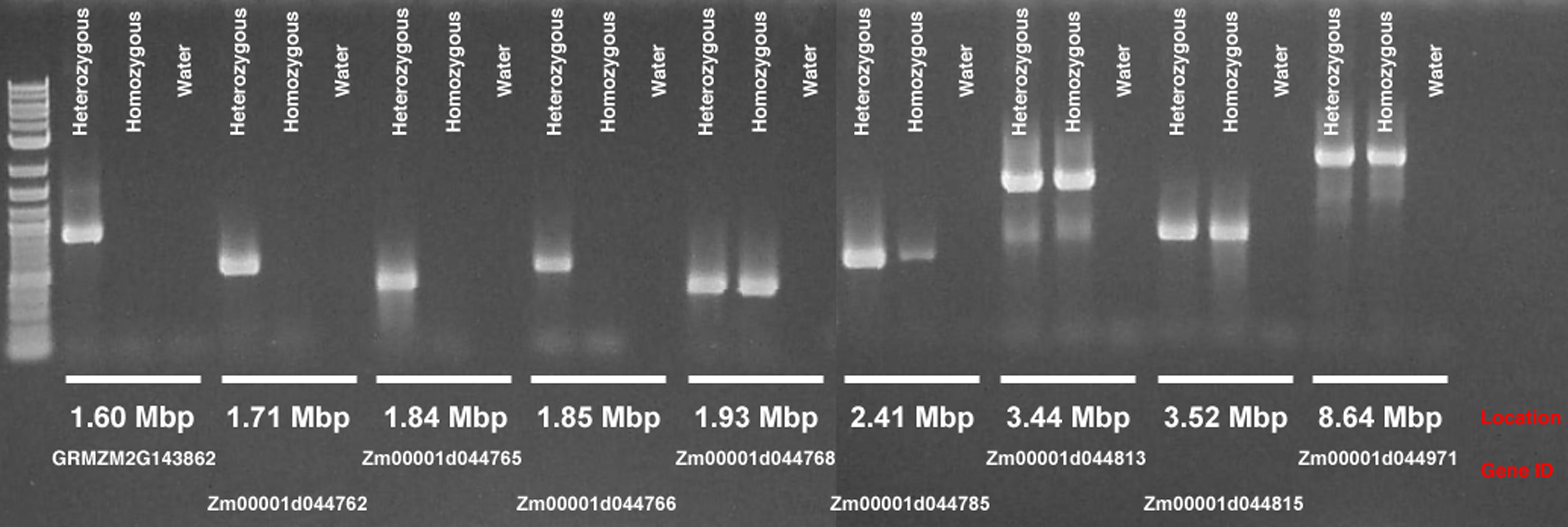


**Supplemental Figure 3.** Mapping of the 9Bic-1 breakpoint using PCR.

Genomic DNA were extracted from 9-Bic-1 homozygous and 9-Bic-1 heterozygous plants. Primers were designed to amplify genes in the distal tip region of 9S. Amplicons will be recovered from heterozygotes, but not from homozygotes, for genes that are positioned distal to the 9-Bic-1 breakpoint. Both samples can amplify genes that are positioned proximal to the 9-Bic-1 breakpoint. The DNA band of Zm00001d44785 using homozygous 9-Bic-1 is weaker than the amplicon recovered from the heterozygote. This might due to a homologous copy being present elsewhere in the genome. Generally, the breakpoint of 9-Bic-1 could be located to the region between 1.85 Mb and 3.44 Mb.


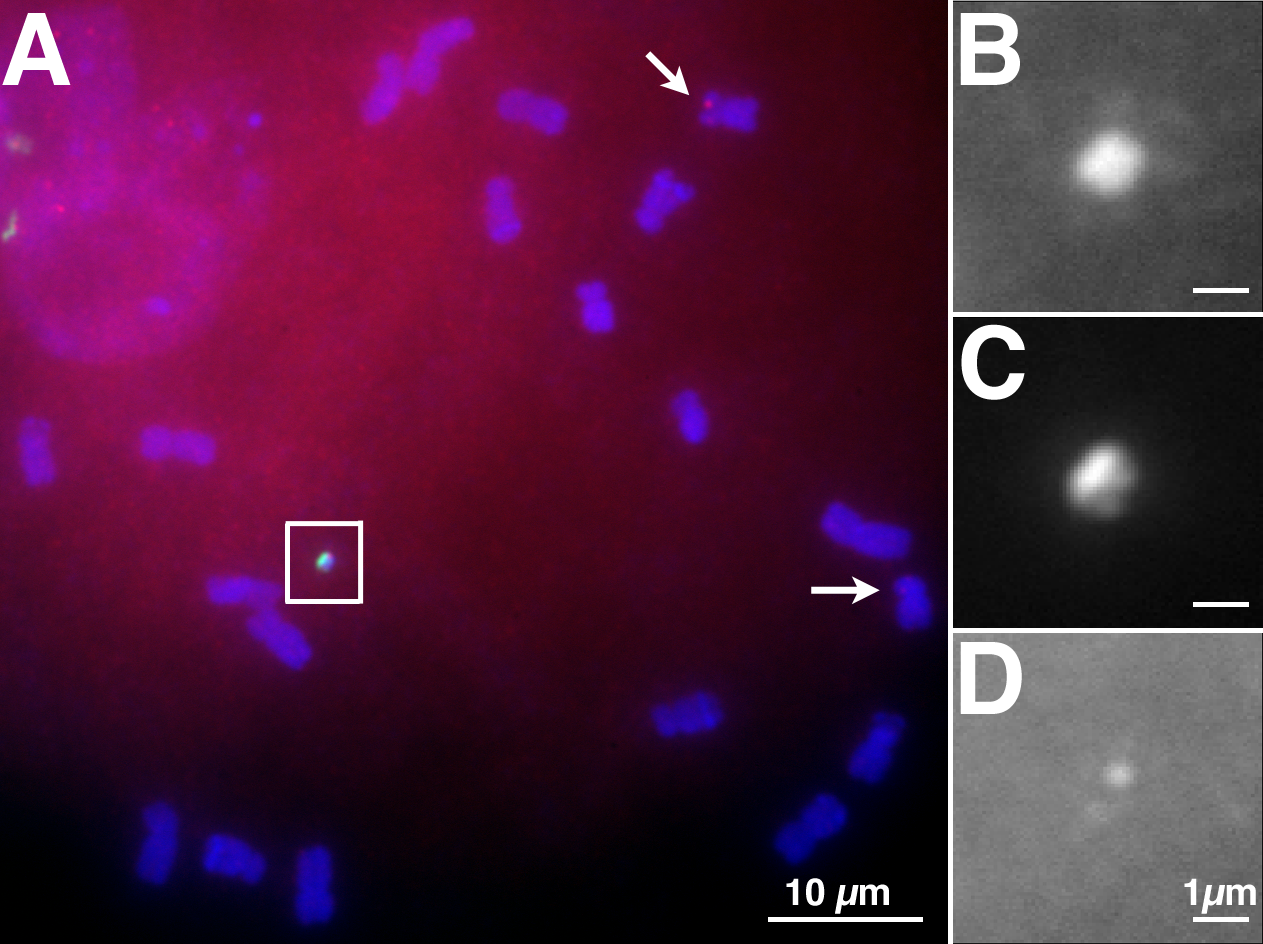


**Supplemental Figure 4.** Mini B496 contains 9S sequences.

Single gene FISH confirmed mini B496 includes a 9S portion (9.51 ~ 9.67 Mb). Mixed probes from two genes region, Zm00001d044996 (B73 chr 9: 9521891-9523869) and Zm00001d045002 (B73 chr 9: 9723241-9738322) were marked as red to hybridize to a root tip spread containing one mini B496. ZmBs (B repeat) was probed as green. **A**, 20 chromosomes plus one mini chromosome were detected. One red signal on the short arm tip of each chromosome 9 is indicated by white arrows. The white box in **A** shows the position of mini B496. Enlarged images of the mini B496 can be seen at **B** (blue channel; DAPI), **C** (green channel; ZmBs) and **D** (red channel; 9S genes).


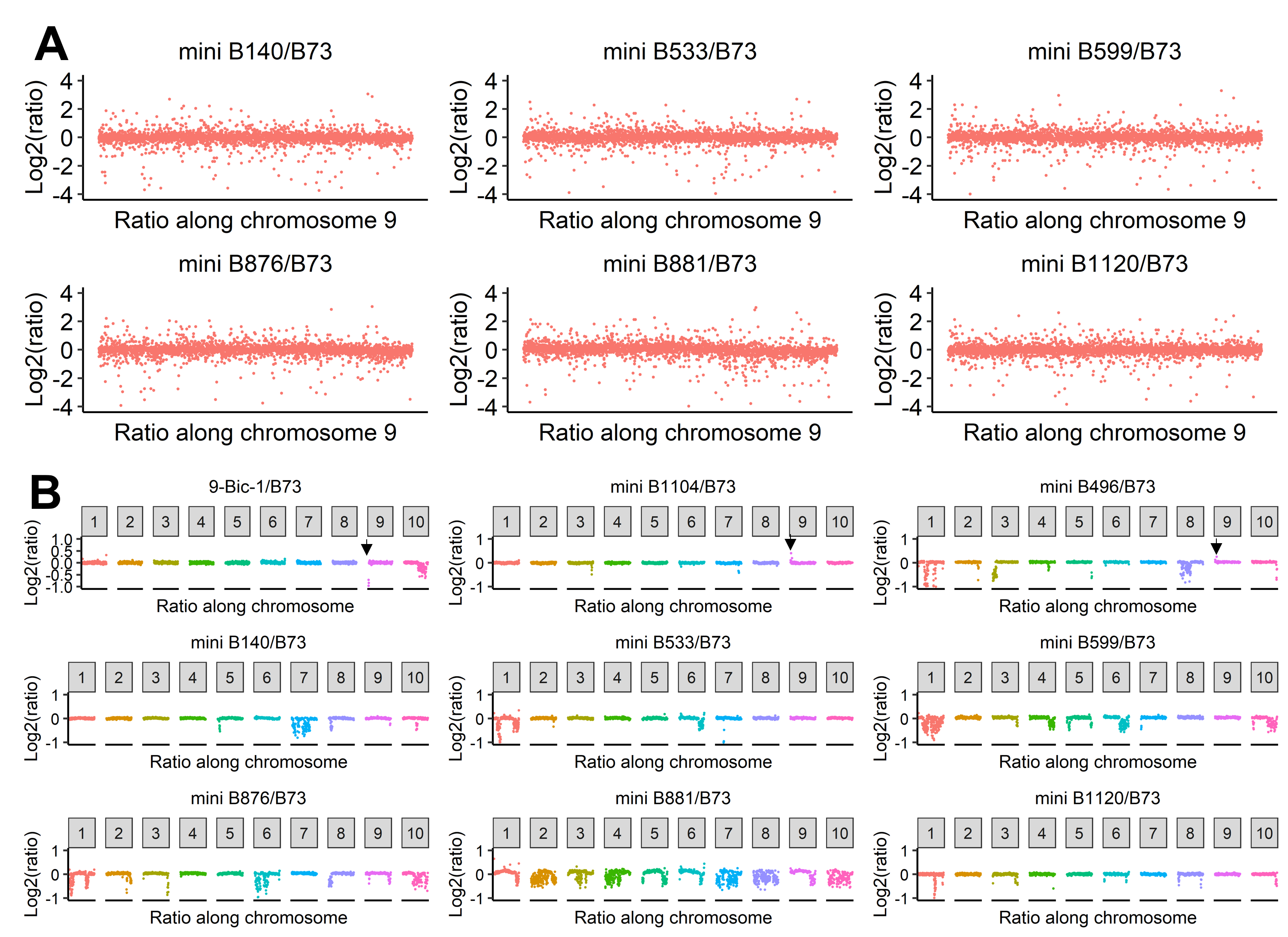


**Supplemental Figure 5.** CNV of mini B chromosomes.

Gene copy number analysis of per gene (**A**) or per 1 Mb region (**B**). All mini chromosomes and the control B73 were subjected to copy number analysis using the read counts from each gene (per gene) or 1 Mb regions (per Mb). In **B**, the ratio changes (black arrow) in 9-Bic-1 (first 3 Mb), mini B1104 (4^th^ Mb), and mini B496 (10^th^ Mb) correspond to per gene ratio changes in Figure 4. The read counts were normalized by RPKM before calculating the ratio. Each data point in **A** and **B** represents one gene and one Mb, respectively, which is plotted along each chromosome on the X axis. The Y axis denotes log ratio of normalized DNA counts with base 2.


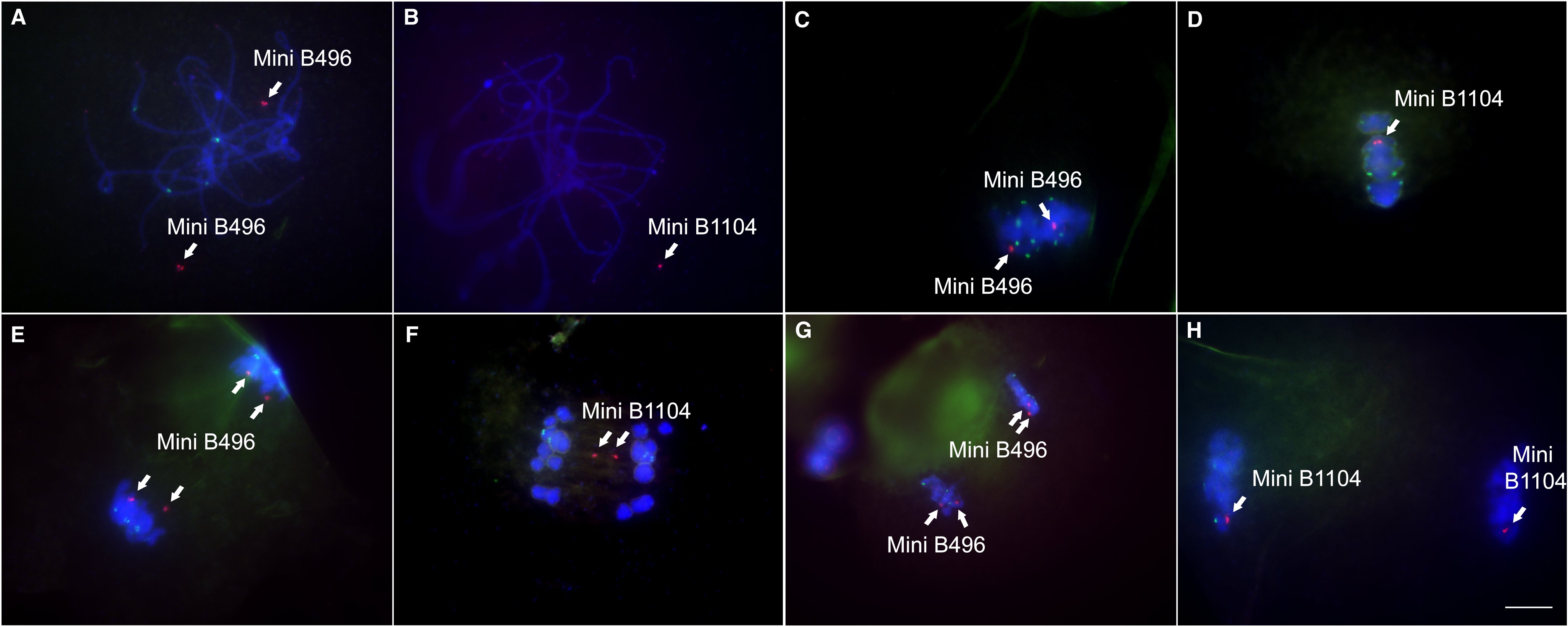


**Supplemental Figure 6.** Cytological analysis of mini B1104 and mini B496.

(**A**) and (**B**) pachytene, (**C**) and (**D**) metaphase I, (**E**) and (**F**) anaphase I, (**G**) and (**H**) telophase I. Mini B496 is shown in **A**, **C**, **E**, **G**; mini B1104 is shown in **B**, **D**, **F**, **H**. The ZmBs is labeled in magenta, and the knob is labeled in green. Blue depicts chromosomes counterstained with DAPI. Note sister chromatid separation and lagging in anaphase I (**E**, **F**). Bars 10 µm.


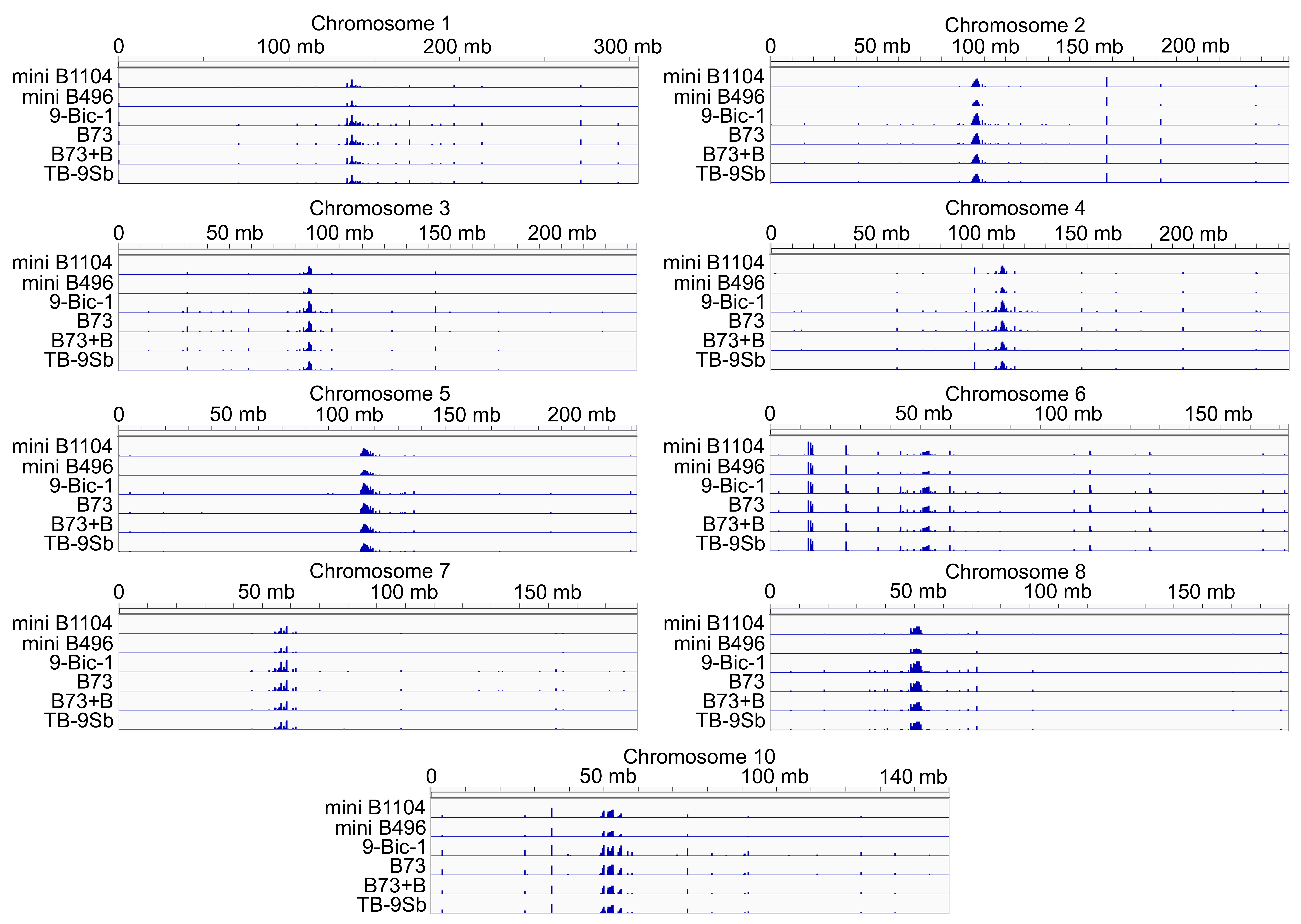


**Supplemental Figure 7.** CENH3 binding for the A centromeres.

CENH3 ChIP-seq reads from six samples were mapped to the B73 genome sequence to identify CENH3 binding on each A centromere.

| **Supplemental Table 1**. Fate of broken chromosomes | | |
| --- | --- | --- |
|  |  |  |
| Type of broken chromosome | Number Observed | % of occurrence |
| Non-heritable | 1391 | 98.51 |
| Translocation | 1 | 0.07 |
| Isochromosome | 10 | 0.71 |
| Heritable minichromosome | 10 | 0.71 |
| Total | 1412 | 100.00 |

**Supplemental Table 1.** Number of broken chromosomes checked/fates of broken chromosomes.

Frequency of kernels and pollen resulting from nondisjunction of 9-Bic-1

| **Supplemental Table 2.** Sequence for primer set used in this study | |  |  |
| --- | --- | --- | --- |
| **9-Bic-1 breakpoint mapping primers** |  |  |  |
| Name | Sequence | Tm | Predicted Size |
| 9s39-1F (Zm00001d044762) | CTTGTCTGTTGCTTGTCCGA | 60.02 | 531 |
| 9s39-1R (Zm00001d044762) | CCAACCGACGGTGAGTAGAT | 59.99 |  |
| 9s41-1F (Zm00001d044765) | AGGAGGCCACAAGAGCAGTA | 60.01 | 403 |
| 9s41-1R (Zm00001d044765) | TGCATACGTACCTCAGAGCG | 60.03 |  |
| 9s43-1F (Zm00001d044768) | GTCGTCGTCGCCTCATTTAT | 60.1 | 400 |
| 9s43-1R (Zm00001d044768) | GAGGTACGTCACCAGGTTCG | 60.57 |  |
| 9s47-1F (Zm00001d044785) | CAACCTACCCTAACAGGCCA | 59.99 | 537 |
| 9s47-1R (Zm00001d044785) | CACTCCCCTTTCCCTTCTTC | 60.04 |  |
| 9s42-A-F | CCTCGTGGCCTTCTTCTTCATCAT | 66.8 | 530 |
| 9s42-A-R | CCCCAACAGTTTGAAATAGAGTGGGT | 66.3 |  |
| 9s33-1F (GRMZM2G143862) | TTGCTGCGAAGAAAGAAACC | 60.5 | 886 |
| 9s33-1R (GRMZM2G143862) | CCTGTCTTCCCACTTTGGAG | 59.69 |  |
| 9s26-2F (Zm00001d044813) | CTTGTCACTGAAGGTCCAGACG | 62.2 | 1456 |
| 9s26-2R (Zm00001d044813) | CGTCCACAAAGTGTTAACCCAA | 62.04 |  |
| 9s25a-1F (Zm00001d044815) | TTTATCACCAGGGTGAGCTCTAT | 59.17 | 741 |
| 9s25a-1R (Zm00001d044815) | TGAAGATTTGTTTGACACCGA | 59.16 |  |
| 9s12-2F | ACCGACTTTCGATGAGCTTG | 60.4 | 1206 |
| 9s12-2R | TGATAAGCATGATGCGGTGT | 60.1 |  |
| **Mini B496 single gene FISH ( ~10 Mbp on 9S)** | |  |  |
| G824574-F | GTCCCAACAAACTCCTCTCCC | 62.2 | 2010 |
| G824574-R | CAACGAGTAGTGCGCAGAGC | 62.3 |  |
| G087196-F3 | TAGCATTGAACCAGATGGTGG | 60.9 | 2059 |
| G087196-R3 | ACGCTGTTCAAAAATGCTTCC | 61.5 |  |
| **CENH3 ChIP qPCR primers** |  |  |  |
| 18s-F | CCATCCCTCCGTAGTTAGCTTCT |  | 180 |
| 18s-R | CCTGTCGGCCAAGGCTATATAC |  |  |
| CRM2LTR-F1 | ATACCCTTGTGGTCGTCCTCC | 62.4 | 161 |
| CRM2LTR-R1 | ACTCCAATATGGTGGGGTTCC | 62.1 |  |
| CRM2LTR-F2 | GATCGTGACAGCCATAGGAGG | 61.9 | 126 |
| CRM2LTR-R2 | TCTTTCGATGAGAGGGTGTGG | 62.5 |  |
| CRM2LTR-F3 | ATCAGGGAGAAGACCGAAAGG | 61.8 | 120 |
| CRM2LTR-R3 | GTGCTTGAACATTCCAATCCG | 62.7 |  |

**Supplemental Table 2.** Primers used in this study.

**References to Supporting Information**

Blavet N, Yang H, Su H, Solansky P, Douglas RN, Karafiatova M, Simkova L, Zhang J, Liu Y, Hou J, Shi X, Chen C, El-Walid M, McCaw ME, Albert PS, Gao Z, Zhao C, Ben-Zvi G, Glick L, Kol G, Shi J, Vrana J, Simkova H, Lamb JC, Newton KJ, Dawe RK, Dolezel J, Ji T, Baruch K, Cheng J, Han F, Birchler JA, Bartos J (2021) Sequence of the supernumerary B chromosome of maize provides insight into its drive mechanism and evolution. Proc Natl Acad Sci, USA 118:e2104254118
